# Bilateral Ankyloblepharon Filiforme Adnatum: A Case Report Highlighting the Importance of Early Recognition and Treatment

**DOI:** 10.7759/cureus.102919

**Published:** 2026-02-03

**Authors:** Chiaki Nakamura, Takashi Negishi, Megumi Ito, Chihiro Koiwa, Shintaro Nakao

**Affiliations:** 1 Department of Ophthalmology, Juntendo University School of Medicine, Tokyo, JPN; 2 Department of Ophthalmology, Nippon Medical School Musashi Kosugi Hospital, Kawasaki, JPN

**Keywords:** ankyloblepharon filiforme adnatum, critical period, deprivation amblyopia, early surgical intervention, visual development

## Abstract

Ankyloblepharon filiforme adnatum (AFA) is a rare congenital anomaly characterized by bands of tissue connecting the upper and lower eyelids, which can lead to visual deprivation and subsequent amblyopia if left untreated. We report a case of a one-month-old girl who presented with bilateral eyelid adhesions causing restricted eyelid opening. The patient was born at 39 weeks of gestation with no prenatal complications. Physical examination revealed tissue bands connecting the eyelids bilaterally, with more severe restriction on the left side. Comprehensive systemic evaluation ruled out associated syndromic conditions, confirming isolated AFA. Due to the risk of deprivation amblyopia during the critical period of visual development, surgical separation of the eyelid adhesions was performed under general anesthesia at three months of age using electrocautery. Histopathological examination confirmed keratinized stratified squamous epithelium. Postoperatively, significant hyperopia and anisometropia were noted, which showed a myopic shift at two-month follow-up. Both eyelids remained well separated with good fixation and tracking in both eyes. This case emphasizes the critical importance of early recognition and prompt surgical intervention in AFA to prevent visual developmental complications. Timely management is essential to avoid stimulus deprivation amblyopia and ensure optimal visual outcomes, particularly in bilateral or extensive cases where the risk of visual impairment is heightened.

## Introduction

Ankyloblepharon filiforme adnatum (AFA) is a rare congenital anomaly characterized by one or more bands of tissue connecting the upper and lower eyelids [1]. The developing eyelid margins remain fused until the fifth gestational month, but may not be completely separated until the seventh month [1,2]. However, in AFA, partial or complete fusion persists, resulting in restricted eyelid opening. While AFA may occur as an isolated finding, it can also be associated with multisystem syndromes such as ankyloblepharon-ectodermal dysplasia-clefting (AEC) syndrome, which is characterized by eyelid adhesions, ectodermal dysplasia, and cleft lip and palate [3,4]. Therefore, when AFA is identified in a neonate, thorough systemic evaluation is warranted to rule out associated anomalies [5].

Infants need clear and unobstructed vision for their eyes and brain to develop properly, especially during the first few years of life when the visual system is most sensitive to stimulation [6]. Visual deprivation during the critical period, which extends from several months to seven or eight years of age, can result in deprivation amblyopia [6]. The severity is greater with unilateral obstruction due to interocular competition [7]. Early surgical intervention to separate the eyelids is crucial in preventing amblyopia during this sensitive period when deprivation is most effective in causing visual deficits [6]. Prolonged eyelid adhesion can lead to stimulus deprivation amblyopia, which may have devastating effects on visual development [8].

Here, we report a case of congenital AFA in a one-month-old girl who presented with restricted eyelid opening in both eyes. After a comprehensive systemic evaluation ruled out associated anomalies, surgical separation of the eyelid adhesions was performed under general anesthesia at three months of age. This case highlights the importance of early recognition and timely management of AFA to prevent visual developmental complications and optimize visual outcomes.

## Case presentation

A one-month-old girl was referred to our department due to restricted eyelid opening in both eyes, noted since birth. She was born at 39 weeks of gestation by spontaneous vaginal delivery, with a birth weight of 3,426 grams. There were no prenatal abnormalities in either the fetus or the mother. The patient had no notable past medical history or family history.

**Figure 1:**
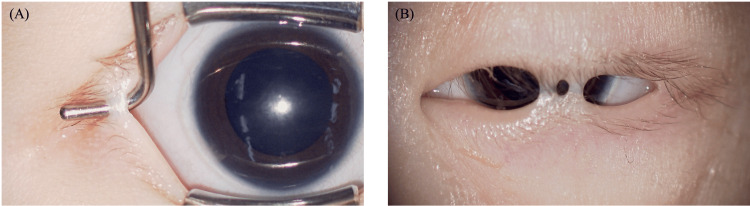
Preoperative findings under general anesthesia (A) The right eye and (B) the left eye are shown. Two areas of band-like tissue connecting the upper and lower eyelids were observed in the center of the left eyelid and one area on the lateral side of the right eye.

On initial examination, a band of tissue connecting the upper and lower eyelids was observed in the left eye, extending from the central to the temporal region. This resulted in restricted eyelid opening, although a corneal light reflex was visible through the gap. The right eye had a similar band on the temporal side, but eyelid opening was relatively well preserved (Figure 1).

Slit lamp and fundus examinations revealed no other abnormalities in either eye. A comprehensive systemic evaluation by the pediatric department revealed no external malformations or developmental abnormalities, ruling out associated syndromic conditions. Due to the risk of deprivation amblyopia, semi-urgent surgical intervention was planned. However, the procedure was postponed due to the patient’s temporary illness, and surgical separation of the eyelid adhesions was ultimately performed under general anesthesia at three months of age.

Intraoperatively, the band-like tissues were successfully divided using electrocautery (Video 1).

**Video 1:** Surgery performed under general anesthesia The band-like tissue was successfully excised using an electrosurgical knife.

**Figure 2:**
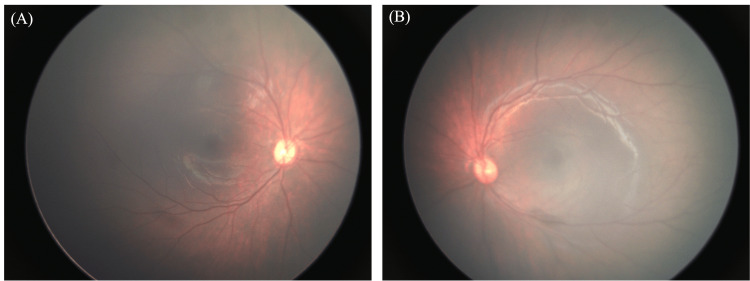
Preoperative fundus findings under general anesthesia (A) Fundus photograph of the right eye. (B) Fundus photograph of the left eye. No abnormalities were observed in either eye.

Fundus examination under anesthesia showed no abnormalities in either eye (Figure 2).

Cycloplegic refraction (with tropicamide and phenylephrine) revealed significant hyperopia and anisometropia: right eye (sphere +5.00 D, cylinder -3.75 D, axis 14°) and left eye (sphere +10.25 D, cylinder -4.00 D, axis 22°). However, these values were considered to be strongly influenced by artifacts caused by the eyelid retractor.

Histopathological examination of the excised tissue showed keratinized stratified squamous epithelium. At the two-month postoperative follow-up (five months of age), both eyelids remained well separated, and fixation and tracking were good in both eyes. Cycloplegic refraction with 1% atropine sulfate eye drops at this time showed a myopic shift: right eye (sphere -1.00 D, cylinder -0.25 D, axis 1°) and left eye (sphere -1.75 D, cylinder -0.50 D, axis 106°). These values were measured during natural palpebral opening without the use of an eyelid speculum, confirming that the intraoperative values were significantly affected by measurement artifacts. Therefore, no glasses were prescribed, and the patient was followed without intervention.

## Discussion

This case underscores the critical importance of prompt recognition and timely surgical intervention in infants with AFA to prevent visual deprivation and optimize developmental outcomes. Early diagnosis and management were pivotal in our patient, allowing for normal visual development and averting the risk of deprivation amblyopia. Previous reports have emphasized that AFA, though rare, may present either as an isolated finding or in association with multisystem syndromes such as AEC syndrome [3,9]. In our case, comprehensive systemic evaluation excluded any syndromic association, consistent with other reports of isolated AFA [10,11]. However, the literature emphasizes the importance of early intervention to avoid the development of amblyopia, even in isolated cases where AFA can present as a solitary malformation requiring immediate attention to prevent functional visual consequences [5]. Visual system vulnerability is particularly pronounced during critical periods in early development, with the visual system being especially susceptible to deprivation during the first years of life [6]. Research demonstrates that the critical period for various visual functions depends on the anatomical level of the visual system being affected, with monocular deprivation having different effects at different developmental stages [6]. Our approach, involving early surgical separation of the eyelid adhesions at three months of age, aligns with these recommendations and supports the notion that timely management is essential to prevent irreversible visual impairment.

Furthermore, while some cases of AFA may resolve spontaneously or be managed conservatively, most authors advocate for early surgical separation, especially when the adhesions are extensive or bilateral [11]. The literature emphasizes that treatment delay can lead to stimulus deprivation amblyopia, which can have devastating effects on visual development [10]. In our patient, the bilateral nature and significant restriction of eyelid opening warranted prompt surgical intervention under general anesthesia. This decision is supported by previous case reports suggesting that delayed management can result in persistent visual deficits and poor visual outcomes [9].

Despite the favorable outcome observed in this case, it is important to acknowledge that visual development in infants with AFA may be influenced by multiple factors beyond mechanical eyelid obstruction alone. Refractive status, binocular interaction, and fixation behavior represent additional and independent contributors to amblyopia risk during early childhood. In this patient, postoperative cycloplegic refraction demonstrated a mild and relatively symmetric myopic shift, which was managed conservatively with close follow-up, considering the absence of significant anisometropia and appropriate fixation behavior as part of the normal emmetropization process. Nevertheless, careful longitudinal monitoring of visual function and refractive development remains essential, as amblyopia prevention requires a comprehensive approach extending beyond surgical correction of the eyelid adhesions.

## Conclusions

AFA is a rare congenital eyelid anomaly that, if left untreated, can result in significant visual developmental complications due to stimulus deprivation. This case highlights the necessity of early recognition, thorough systemic evaluation to exclude associated syndromes, and prompt surgical intervention, especially in cases with bilateral or extensive adhesions. Timely management is essential to prevent deprivation amblyopia and ensure optimal visual outcomes. Clinicians should remain vigilant for this condition in neonates with restricted eyelid opening and intervene without delay to safeguard normal visual development.
